# Comparison of Polyaromatic Hydrocarbon Residue Concentrations in *Clarias gariepinus* Smoked with Traditional and Mechanical Kilns

**DOI:** 10.5696/2156-9614-10.28.201215

**Published:** 2020-12-07

**Authors:** Oluseun Osineye, Ayo Jesutomi Abiodun-Solanke, Elizabeth Mangai, Ebele Okeke, Bethel Jahnezim

**Affiliations:** Fisheries Technology Department, Federal College of Fisheries and Marine Technology, Victoria Island, Lagos, Nigeria

**Keywords:** kilns, fish, smoking, PAHs, concentrations

## Abstract

**Background.:**

Wood, a common fueling material for fish smoking in Nigeria, contains polycyclic aromatic hydrocarbons (PAHs) which have been found to be carcinogenic and pose a human health hazard.

**Objectives.:**

The present study investigated the interactions and effects of kiln types on levels of PAHs in smoked fish.

**Methods.:**

Twenty fresh samples of *Clarias gariepinus* with an average size of 800 g were eviscerated, washed and salted for smoking. Sixteen (16) of the fish were randomly and equally allotted to each of the two kilns (treatments) and replicated three times in a completely randomized design. Each kiln was loaded and heated up with hard wood charcoal and the effects were evaluated on the resulting smoked fish. The remaining four fish samples were kept under refrigeration (controls). Samples of the smoke-dried fish from each kiln were homogenized using a porcelain mortar and pestle, sieved through a 250-μm^2^ sieve, and packaged in labeled airtight containers prior to extraction. Pure extracts from the samples were subjected to gas chromatography.

**Results.:**

The results showed that the PAH concentration in non-smoked catfish was 1.0 mg/kg, the PAH concentration in fish samples smoked with a traditional kiln was 2.0 mg/kg, and no PAHs were detected in samples smoked with a mechanical kiln.

**Conclusions.:**

The level of PAH contamination of smoked fish is dependent on the type of kiln used, as demonstrated by the differences between fished smoked with traditional and mechanical kilns in the present study.

**Competing Interests.:**

The authors declare no competing financial interests

## Introduction

Nigeria's high ambient temperature, relative humidity and inadequate facilities for processing and storage encourage fish damage and spoilage, leading to enormous waste. To curb post-harvest losses and ensure supply throughout the year, post-harvest processing and preservation of fish are important to prolong the shelf storability of fish and ensure a sustainable off-season supply, with increased profit for fish farmers or fisherpeople.^1^

The term “fish processing” refers to the procedures related to fish and fish products from the time of catch or harvest, and conversion to products until the final product reaches the customer. Processing and preservation of fish is important as fish deteriorate rapidly after harvest and there is a need to prevent losses after harvest.^2^ Fish preservation is a crucial aspect of fishery commerce. Fish are preserved using such methods as smoking, freezing, heat treatment (sterilization, pasteurization, etc.) and drying, in order to extend its shelf life.^3^ According to the Food and Agriculture Organization (FAO) 2001, fish and fish products can be preserved by a number of methods, including temperature control via freezing, refrigerating or icing; control of water activity (a_w_) through the processes of salting, drying, freeze-drying or smoking; withdrawal of oxygen by vacuum packing; using microwave heating or ionizing radiation to physically control the levels of microorganisms; and the addition of acids to chemically control microbial loads.^4^

When smoke is applied to foodstuffs, the process is basically physical and based on different phenomena like diffusion, absorption, and dissolution as well as deposition in force fields.^5^ It is accompanied by a chemical process where smoke compounds interact with food components.^6^ The most commonly used material to produce smoke is wood. It is comprised of diverse polymer groups of lignin, pectin, cellulose, and hemicellulose. These four groups of wood polymersare believed to be partially connected to each other through chemical bonds.^7^ Wood smoke contains a number of compounds that form when the components of wood undergo pyrolysis. More than 300 substances have been detected, and many more exist.^8^ Many of these smoke components are present in smoked foods. The most important classes of chemical compounds detected in smoke and liquid smoke preparations are phenols, furans, carbonyls, polycyclic aromatic hydrocarbons (PAHs), lactones, alcohols and esters.^9^ Smoking confers desirable outcomes on foods including flavoring, coloring, and preservation. However, undesirable effects occur when toxic constituents of smoke contaminate food or when essential amino acids produced from food proteins are destroyed as in certain classes of smoke and liquid smoke.^10^ Certain carbonyls, acids and phenols are primarily responsible for the typical aroma of smoked foods. These compounds also cause, at least in part, different flavors in smoked foods.^10^

The term PAH refers to neutral, non-polar molecules present in fuels from fossils (oil and coal) and deposits from tars, which form when inadequate amounts of oxygen or other factors cause the partial combustion of organic matter (like in engines, incinerators or in fires that occur in forests where biomass combusts).^11^ High quantities of PAHs are produced in cooked foods, as in meat processed over open fires at high temperatures. An example of a PAH produced by coal tar is the highly carcinogenicbenzo[a]pyrene which contains mutagenic metabolites.^12^ Benzopyrenes form when monocyclic benzene and tetracyclic pyrene rings fuse, resulting from partial combustion at temperatures reaching 300–600°C (572–1,112°F).^13^

As the toxic constituents of curing smoke and smoke condensates, PAHs like benzo(a)-pyrene have received great attention since some of these substances contain carcinogenes.^9^ However, certain phenols are classified as toxic since they produce a co-carcinogenic effect in the presence of PAHs.^11^ Polycyclic aromatic hydrocarbons are affected by many factors of smoke generation such as temperature of combustion, oxidation, air supply, density, length and temperature of the smoke cure, characteristics of the product surface and composition.^14^

The use of a kiln to process fish through hot smoking has long been practiced traditionally worldwide. A smoking kiln is a thermally insulated oven with a chamber where adequate temperatures are produced to carry out processes such as hardening, drying or chemical changes.^15^ Many types of smoking kilns are employed in fish smoking and these can be classified under two broad categories: traditional kilns and mechanical kilns.

Traditional kilns used for smoke-drying are of very simple design and construction. In Africa, the types of available kilns used for smoking include the traditional open fire, cylindrical drum, mud brick and brick.^3^ The traditional kiln, most often utilized for fish smoking is either rectangular or cylindrical in shape and constructed with metal or mud. Recently, mechanical kilns have come to be preferred to traditional types for preserving fish since these new kilns require less processing time and result in better product quality among other benefits.

Diverse models of improved smoking kilns have been developed in different parts of Africa with improvements aimed at producing high quality fish with uniform heat distribution.^16^ They are equipped with devices for controlling interactions between smoke and fish. Such smoking kilns allow the air velocity, temperature and humidity to be uniform and also offer protection of the environment, improved economy of operation and high sanitation standards.^3^ Mechanical smoking kilns are expected to result in better quality products, be more acceptable to consumers and can be designed in Nigeria with locally available materials. Mechanical smoking kilns could be adapted for use in rural areas where electric power is not yet commonly used.

The present study aimed to evaluate the quantities of PAH deposited by charcoal on smoked fish compared with wood, and compared the effect of different kilns on PAH concentrations in smoked fish.

## Methods

The present study was conducted in March 2019 at the Federal College of Fisheries and Marine Technology Victoria Island, Lagos. Nigeria.

The traditional smoking kiln (open-drum type) used in this study consists of fish trays which are containers into which the fish are loaded and smoked. It is made of galvanized wire trays. The trays are placed on top of the drum kiln, which has a wooden frame. The kiln has the capacity of carrying five (5) trays loaded with fish.This container is used for burning charcoal that produces the heat used for smoking the fish. The traditional smoking kiln also has a charcoal box produced from metal sheets. It is inserted and retrieved through a fireplace cut out from the drum sheet.The kiln body is made of drum sheets hammered into a rectangular shape. It has the dimension of 1 m by 1.5 m by 1 m. The oil drum sheet kiln body is meant to allow upward passage of smoke from the fireplace to the fish trays.

The mechanical smoking kiln features some improvements over the traditional kiln as shown in Figure 2.

**Figure 1:**
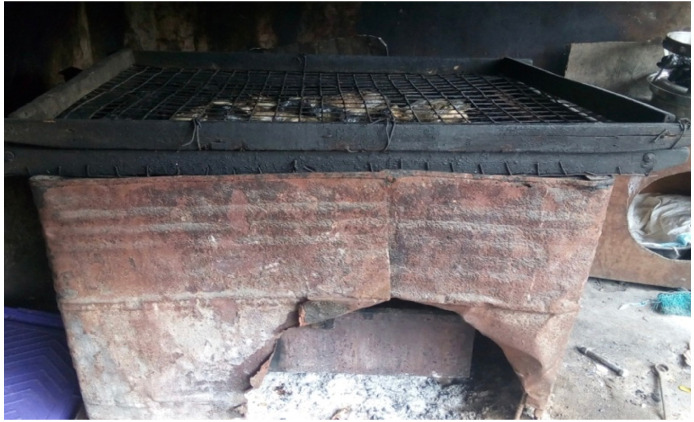
An open-drum traditional kiln

**Figure 2:**
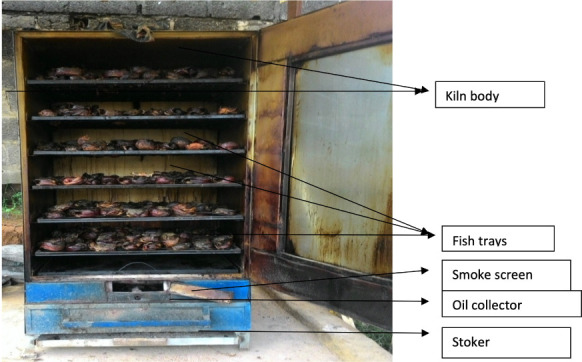
A mechanical kiln

The kiln has six (6) movable tray shelves on which the fish are loaded for smoking. The charcoal is loaded into a fire box with an ash collector situated just below the box. The smoking kiln used was rectangular in shape with dimension of 1 m × 1 m × 2 m. It was designed and constructed using locally available materials. Its inner lining was made of galvanized iron sheet insulated with asbestos particles. The double wall structure with insulating material functions to conserve heat energy by reducing heat loss and the smooth inner chamber allows unrestricted flow of the smoke aerosol to process the fish.

A very important feature of the mechanical kiln is the perforated damper/oil collector- a metal sheet directly above the stoker, which helps to prevent the oil extracted from the fish during smoking from dropping into the fire place and accumulated oil was drained regularly. The most important improvement of this kiln is the ability of the metal sheet to screen off smoke and fire from getting to the fish being smoked. The smoke was let out through an opening in the fire box.

### Sample collection and preparation

Twenty (20) fresh samples of African catfish with an average size of 800 g were procured from Akin-Sateru farm, Lagos State. The fresh fish were subjected to low temperature storage (refrigeration) at 15°C for slow and effective rigor mortis and better conversion of fish muscle. The fresh fish were eviscerated by napping (cutting through the fish flesh between the head and the belly region)to give room for total removal of the viscera. The fish were then washed thoroughly and salted evenly, bearing in mind the importance of uniform treatment to avoid bias, in preparation for smoking. Both kilns (traditional and mechanical) were loaded with the same quantity and heated up simultaneously with hardwood charcoal as fuel. Four of the fresh fish were kept in a refrigerator to serve as controls, while an equal number of fish were randomly transferred into and smoked in each kiln simultaneously (eight (8) fish per kiln) under the same condition. The fish were closely monitored until they were fully dried. A porcelain mortar and pestle were scrubbed, rinsed with warm water and allowed to drip dry. Samples of dried fish from each kiln were homogenized using the porcelain mortar and pestle, sieved through a 250-μm^2^ sieve and packaged in labeled air-tight containers prior to extraction.

### Extraction

The extraction of PAHs was performed using the method detailed by Pena *et al*. (2006).^17^ Two grams (2 g) of dried fish sample from each kiln and 2 g of fresh fish sample (control) were weighed into different labeled and cleaned 50-ml beakers used as extraction containers. Each batch was analyzed in duplicate. Ten (10) ml of dichloromethane used as the extraction solvent was introduced into the sample, which was properly mixed and left to settle. The sample was cautiously filtered into a clean extraction bottle that was pre-rinsed with the solvent by using filter paper fitted into a Buchner funnel. Concentration of the extracts into 2 ml was performed and this was then conveyed for the purification/separation step.

### Purification/separation

The base of the chromatographic column was fitted with 1cm of moderately packed glass wool. A slurry preparation of 2 g activated silica in 10 ml dichloromethane was placed into the chromatographic column in order to serve as the stationary phase to absorb the impurities so that the pure sample can elute through the phase. Next, 0.5 cm^3^ of sodium sulphate was introduced as a drying agent to the top of the column to remove water from the organic extract. A further 10ml of dichloromethane was used to rinse the column which was pre-eluted with 20 ml of dichloromethane and served as the mobile phase that carried the pure sample down through the stationary phase. This moved through the column at 2 ml/min until the liquid in the column rested just on top of the sulphate layer. Instantly, some of the extracted samples (1 ml) was moved into the chromatographic column while the bottle used for extraction was rinsed with 1ml of dichloromethane and introduced into the column. The element was collected using a 10 ml graduated cylinder by opening the stop cork of the column. Just prior to exposing the sodium sulphate layer to air, two increments of dichloromethane were added to the column. Precisely 10 ml of the eluent was measured, collected, and labeled.

### Analysis of polycyclic aromatic hydrocarbons by gas chromatography

For gas chromatographic analysis, the concentrated aliphatic fractions were moved into previously labeled glass vials having rubber clip caps. The Hewlett Packard 5890 series II gas chromatography apparatus, fitted with a flame ionization detector (FID) (Hewlett Packard, Wilmington, DE, USA) and powered with Hewlett Packard Chem Station Rev. A 09:01 (10206) software was used to identify and quantify the compounds. A fused silica column [30 m*0.25 μm film of HP-5 (thickness)] was used; with the inlet and injection temperature set at 275–310°C. A split ratio of 8:1 was adopted for split injection and using hypodermic syringe, and 1 μl of the concentrated sample was injected into the column through a rubber septum. Area of separation occurrences were noted and used in detecting the sample with the aid of a flame ionization detector. The temperature of the column was programmed as follows: holding at 65°C for 2 min; 65–260°C at 12°C/min; 260–320°C at 15°C /min and maintained at 310°C for 8 minutes, with an oven temperature set at 65°C. Nitrogen was used as a carrier gas. Hydrogen and compressed air pressure were measured at 30 psi and the oven was programmed at an initial temperature of 65°C. Verification of peaks was done based on retention times compared to those of external PAHs.

## Results

There were only four PAHs detected in the differently treated fish samples.As shown in Table 1, the controls had a total of 1 mg/kg PAHs, with 0.5 mg/kg each of naphthalene and phenanthrene. Fish smoked in a traditional kiln had a total of 2 mg/kg PAHs, with 1.0 mg/kg of phenanthrene and 0.5 mg/kg each of fluoranthene and benzo(a)pyrene. The mechanical kiln smoked fish had no detectable PAHs.

**Table 1:** Average Concentrations (mg/kg) of Polycyclic Aromatic Hydrocarbons (PAHs) in Catfish by Kiln Type

PAHs	Control	Traditional kiln	Mechanical kiln
Naphthalene	0.50	0.00	0.00
Phenanthrene	0.50	1.00	0.00
Fluoranthene	0.00	0.50	0.00
Benzo(a)pyrene	0.00	0.50	0.00
*Total PAHs*	*1.00*	*2.00*	*0.00*
*Carcinogenic PAHs*		*0.50*	*0.00*
*Non-Carcinogenic*	*1.00*	*1.50*	*0.00*

Table 2 presents the descriptive treatment of PAH concentrations. The means of the PAHs were collated and analyzed.

**Table 2:** Descriptive Treatment of Polycyclic Aromatic Hydrocarbon (PAH) Concentrations

	N	Mean	SD	SE	95% Confidence Interval for Mean		Min	Max

					Lower bound	Upper bound		
Control	16	.0625	.17078	.04270	-.0285	.1535	.00	.50
Traditional	16	.1250	.28868	.07217	-.0288	.2788	.00	1.00
Mechanical	16	.0000	.00000	.00000	.0000	.0000	.00	.00
Total	48	.0625	.19638	.02834	.0055	.1195	.00	1.00

Abbreviations: SD, standard deviation; SE, standard error

Table 3 presents the one-way ANOVA of the PAH concentrations. A p-value of 0.2 indicates that there was no significant difference between the groups of fish samples.

**Table 3:** One-way Analysis of Variance (ANOVA) of Polycyclic Aromatic Hydrocarbon (PAH) Concentrations

Concentrations	Sum of Squares	df	Mean Square	F	Sig
Between groups	.125	2	.063	1.667	.200
Within groups	1.687	45	.037		
Total	1.812	47			

Abbreviations: df, degrees of freedom. Sig, significance

## Discussion

In the past, according to the Codex Alimentarius Commission, benzo(a)pyrene was listed as the only carcinogenic PAHs to be aware of,^18^ with a permissible level of 2 mg/kg. PAH indicators lists were revised in 2010 and six added: (benz(a)anthracene, benzo(b)fluoranthene, benzo(k)fluoranthene, chrysene, dibenzo(ah)anthracene, and indeno(1,2,3-cd) pyrene with the permissible limit of 10 mg/kg.^19^

The PAHs listed above are classified as carcinogenic PAHs. The PAH values in the control samples fell under the limit for safe consumption. The result of the present study also revealed the possibility of the presence of PAHs in a number of water bodies, as observed by the International Agency for Research on Cancer (IARC),^18^ and this could be a function of the chemical nature of underlying rocks, vegetation, aquatic life, interactions of water bodies with other water bodies, as well as the influences of other biotic and abiotic factors surrounding the water bodies.^19^ This also indicates the water in which the catfish were cultured was free of pollution by substances that are sources of PAHs. The results of the present study are in agreement with observations made by Stolyhwo and Sikorski that fish and marine invertebrates may naturally contain small or undetectable amounts of different PAHs absorbed from the environment.^5^ The PAH concentrations observed in fish samples smoked with a traditional kiln were also low (total= 2.0 mg/kg) compared with higher values of 17.0±4.6 mg/kg reported by Hokkanen *et al.* where wood was used.^20^ Phenanthrene was the highest, occurring at a concentration of 1.0 mg/kg, while fluoranthene and benzo(a)pyrene were detected at 0.5 mg/kg each. This concentration showed a slight increase compared with that of the fresh fish samples. There was thus little addition of PAHs into the fish samples during the smoking process and remained under acceptable limits. This indicates that there was no addition of PAHs in the sample smoked with the mechanical kiln during the smoking process. As seen in Table 3, there were no significant differences in PAH concentrations in the differently treated samples. This result affirms the efficiency of the mechanical kiln design in screening off PAHs contained in the fuel source from entering the fish being smoked.

Other factors may also be responsible for the minimal concentration of PAHs detected in all the samples. Differences in kiln design showed no variation in the results, which could be attributed to the charcoal fuel source which has undergone decomposition through anaerobic combustion, thereby screening off most PAHs possibly present in the wood. This is in agreement with the results of Simko (2002) that attributed the primary source of PAHs to wood combustion, especially for smoked fish products.^6^ However, charcoal is a product of destructive distillation of wood during which the volatile constituents of the wood are gassed out. For the present study, both kilns used (traditional and mechanical) were fueled with charcoal. This possibly contributed to the minimal level of PAHs that infiltrated the fish during the smoking process since the fuel source has already been stripped of most of its PAH content.

These results indicate that when charcoal is used as a fuel source, fish smoked with a traditional kiln would be safe for consumption. However, we recommend the use of a mechanical kiln which completely screens off any residual PAHs that may still be present in the charcoal.

Much lower quantities of PAHs are found in charcoal than in wood as a result of wood pyrolysis. Mechanical kilns of various types, including the Nigeria Institute for Oceanography and Marine Research (NIOMR), University of Ibadan (UI) The Conseil Ouest et Centre Africain pour la Recherche et le Développement Agricoles (CORAF) and Federal College of Fisheries and Marine Technology (FCFMT) models, in addition to being great improvements on the traditional kiln, commonly have special features that shield the fish being smoked from smoke directly emanating from the fuel chamber of the kiln. This helps to shield the fish from being contaminated by the PAH content of the smoke.

Through continuous exposure, either in vitro or in vivo, to PAHs, benzo(a)pyrene in particular has been linked to cancers of the skin, lung, bladder and gastrointestinal tract in laboratory animals. Adverse reproductive and developmental effects have also been reported in animal studies.^19^ Cataracts, jaundice, kidney and liver damage are long-term health effects of PAH exposure and symptoms like eye irritation, nausea, vomiting, diarrhea and confusion are short-term health effects that have been reported.^19^ Improved and environmentally friendly energy sources such as briquette and biochar are being developed as alternative energy sources to wood and charcoal, and are hoped to be less hazardous to human health.

## Conclusions

Pyrolysis of hardwood charcoal leads to a low charcoal content of PAHs, leaving almost insignificant residual PAHs levels, minimizing the contamination of fish samples during smoking. Hence, hardwood charcoal has been found to produce safer smoked products with lower PAH levels than wood. The mechanical kiln is a great improvement over the traditional kiln, primarily due to two unique features: a shield that screens the fish being smoked from residual PAH contaminants emanating from the charcoal fuel and a mechanism that amplifies the heat generated to an extent that is capable of subliming (converting hardwood charcoal to gaseous matter) any available PAHs away from the smoked fish. This explains the absence of PAHs in the fish smoked with the mechanical kiln. It is recommended that further studies on mechanical kilns be conducted to identify other features and functions that make mechanical kilns superior to traditional kilns.
